# 46 Integration of Cutaneous Functional Units Principles in Burn Rehabilitation: A Diffusion of Innovations Assessment

**DOI:** 10.1093/jbcr/irad045.020

**Published:** 2023-05-15

**Authors:** Miranda Yelvington, Ingrid Parry

**Affiliations:** Arkansas Children's Hospital, Little Rock, Arkansas; Shriners Hospital for Children, Carmichael, California; Arkansas Children's Hospital, Little Rock, Arkansas; Shriners Hospital for Children, Carmichael, California

## Abstract

**Introduction:**

Early recognition of burn scar contractures can lead to a more targeted and effective therapy regimen, potentially preventing loss of range of motion and improving burn survivors' quality of life. In burn rehabilitation, consideration of the principles of cutaneous functional units (CFUs), or fields of skin as they relate to movement at associated joint, can allow occupational and physical therapists to explain and predict limitations and formulate treatment plans individualized to each patient’s pattern of injury. Evidence-based research demonstrates the potential of CFU principles to improve upon the current standard of care, but the consistent use of these principles has been slow to diffuse through burn care. The Diffusion of Innovations theory, by Everett M. Rogers, provides a framework for the examination of the process by which CFU principles (innovation) have been diffused into clinical burn practice (channel) since its emergence in burn literature (time) within the burn therapy network (social system).

**Methods:**

Using purposive sampling survey invitations were sent to occupational and physical therapists who work in burn care and posted to therapy-related social media groups. Responses were collected and demographic variables and trends were analyzed and are reported here.

**Results:**

Respondents (297) were Occupational Therapist (52%) and Physical Therapists (49%) and were inclusive of all practice areas. The majority of respondents work in a burn unit (81%) in North America (70.7%). Most respondents (78.4%) report familiarity with the term CFU as it relates to burn rehabilitation. Of those familiar, the majority of respondents reported their knowledge (66.7%) and ability to apply (65.7%) at an intermediate level or greater. A slight majority (59.3%) responded that the CFU concepts influenced their performance of burn rehab, while 40.7% said the concepts did not influence their practice. 40-69% of respondents correctly answered CFU knowledge questions but only 15% of respondents correctly completed CFU identification questions. Respondents (77%) report barriers to the use of CFUs including difficulty incorporating into practice, time constraints, and the need for more education. Only 5.9% of respondents endorsed no interest in using CFU concepts.

**Conclusions:**

Survey respondents recognize the relative advantage of using CFU concepts and find them compatible with current values and practices. However, barriers in knowledge and applicability exist, suggesting that current educational resources have not enabled the information to be easily understood and applied. Diffusion can be improved by developing tools to assist therapists in understanding and incorporating CFU principles.

**Applicability of Research to Practice:**

This framework can be used to develop a strategy to enhance CFU diffusion within the burn therapy community.

